# Association between a history of frequent masturbation and anxiety/depression in patients with psychogenic erectile dysfunction

**DOI:** 10.1186/s12610-026-00300-w

**Published:** 2026-01-27

**Authors:** Jinlong Yang, Wenju Wu, Yilin Zhao, Junjie Liu

**Affiliations:** 1 Department of Urology, The Affiliated Hospital of Xuzhou Medical University, Xuzhou, China; 2 The First Clinical Medical College, Xuzhou Medical University, Xuzhou, China

**Keywords:** Frequent masturbation, Psychogenic erectile dysfunction, Anxiety, Depression, Psychological resilience, Masturbation fréquente, Dysfonction érectile psychogène, Anxiété, Dépression, Résilience psychologique

## Abstract

**Background:**

Psychogenic erectile dysfunction patients accounted for a substantial percentage of younger erectile dysfunction ones. This cross-sectional observational study investigated the correlation between a history of frequent masturbation and anxiety/depression symptoms in patients with psychogenic erectile dysfunction .

**Results:**

Baseline characteristics showed significant between-group differences in age (the Frequent Masturbation History group younger, *P* < 0.05), but not in disease duration, residence, or lifestyle factors. The Frequent Masturbation History group demonstrated significantly higher anxiety (GAD-7: Z=-2.17, *P* = 0.030) and depression scores (PHQ-9: Z=-3.01, *P* = 0.003), alongside significantly lower psychological resilience (CD-RISC: Z=-2.53, *P* = 0.011) compared to the Non-Frequent Masturbation History group. These findings indicate that frequent masturbation history in psychogenic erectile dysfunction patients is associated with younger age, elevated anxiety/depression symptomatology, and reduced stress adaptability.

**Conclusions:**

Clinical implications suggest incorporating behavioral pattern assessment and psychological screening into psychogenic erectile dysfunction evaluations. Targeted interventions should focus on cognitive-behavioral therapy to address maladaptive beliefs, mindfulness training to reduce performance anxiety, and partner-involved support to disrupt the observed “masturbation to anxiety/depression to erectile dysfunction” cycle. Future longitudinal studies integrating biopsychosocial assessments are warranted to elucidate temporal relationships.

## Résumé

**Contexte:**

Les patients atteints de dysfonction érectile psychogène sont constitués d’un pourcentage important d’hommes jeunes. Cette étude observationnelle transversale a examiné la corrélation entre un historique de masturbation fréquente et des symptômes d’anxiété/dépression chez des patients souffrant de dysfonction érectile psychogène.

**Résultats:**

Les caractéristiques de base montraient des différences d’âge significatives entre les groupes (le groupe plus jeune Antécédant de Masturbation Fréquente p <0,05), mais pas en termes de durée de la maladie, de résidence ou de facteurs de mode de vie. Le groupe Antécédant de Masturbation Fréquente a montré une anxiété significativement plus élevée (TAG-7 : Z=-2,17, p=0,030) et des scores de dépression (PHQ-9 : Z=-3,01, p=0,003), ainsi qu’une résilience psychologique significativement plus faible (CD-RISC : Z=-2,53, p=0,011) par comparaison au groupe Antécédant de Masturbation non Fréquente. Ces résultats indiquent que les antécédents fréquents de masturbation chez les patients atteints de dysfonction érectile psychogène sont associés à un âge plus jeune, à une symptomatologie accrue de l’anxiété/dépression, et à une adaptabilité réduite au stress.

**Conclusions:**

Les implications cliniques suggèrent d’intégrer l’évaluation des schémas comportementaux et le dépistage psychologique dans les évaluations de la dysfonction érectile psychogène. Les interventions ciblées devraient se concentrer sur la thérapie cognitive-comportementale pour traiter les croyances inadaptées, sur l’entraînement à la pleine conscience pour réduire l’anxiété de performance, et sur le soutien impliqué de la partenaire pour rompre le cycle observé « masturbation par anxiété/dépression jusqu’à la dysfonction érectile ». De futures études longitudinales intégrant des évaluations biopsychosociales sont nécessaires pour élucider les relations temporelles.

## Introduction

Erectile dysfunction (ED) is a common male sexual dysfunction characterized by the persistent inability (lasting at least three months) to attain and maintain sufficient penile erection for satisfactory sexual intercourse. This condition significantly impacts the physical and mental well-being of both the patients and their partners, as well as their quality of sexual life [1]. Epidemiological studies indicate that ED has become one of the most prevalent urological issues among men worldwide. Psychogenic erectile dysfunction (pED), a subtype of ED, is clinically associated not only with erectile difficulties but also with personality traits, psychosocial stress response, anxiety and depression [2]. A study reports that pED patients accounted for a substantial percentage of younger ED ones [3]. Among males under 40 years old, erectile dysfunction (ED) affected 85.2% of the cohort [4].

Current scholarly perspectives on the relationships between masturbation, anxiety, depression, and erectile dysfunction (ED) are inconsistent. A study has reported that among married heterosexual Chinese men, masturbation and associated feelings of guilt are relatively common [5]. Men who prefer masturbation over partnered intercourse show a significantly higher risk of sexual dysfunction [6]. Furthermore, excessive masturbation has been identified as an independent risk factor for the progression from mild to moderate-severe ED [7]. A social-media–based epidemiological survey also indicated that most users attribute ED to psychological factors, excessive pornography consumption, or masturbation [8]. In contrast, other researchers have found that masturbation frequency is only weakly or not significantly associated with erectile function, ED severity, or relationship satisfaction [9]. Additional empirical evidence suggests that masturbation frequency is not correlated with life satisfaction or depressive symptoms [10].

Therefore, this cross-sectional study (an observational study design) aims to investigate the correlation between a history of frequent masturbation and anxiety/depression scores in patients with pED. The findings are intended to provide insights for healthcare professionals in developing targeted interventions.

## Patients and methods

### Study participants

**Fig. 1:**
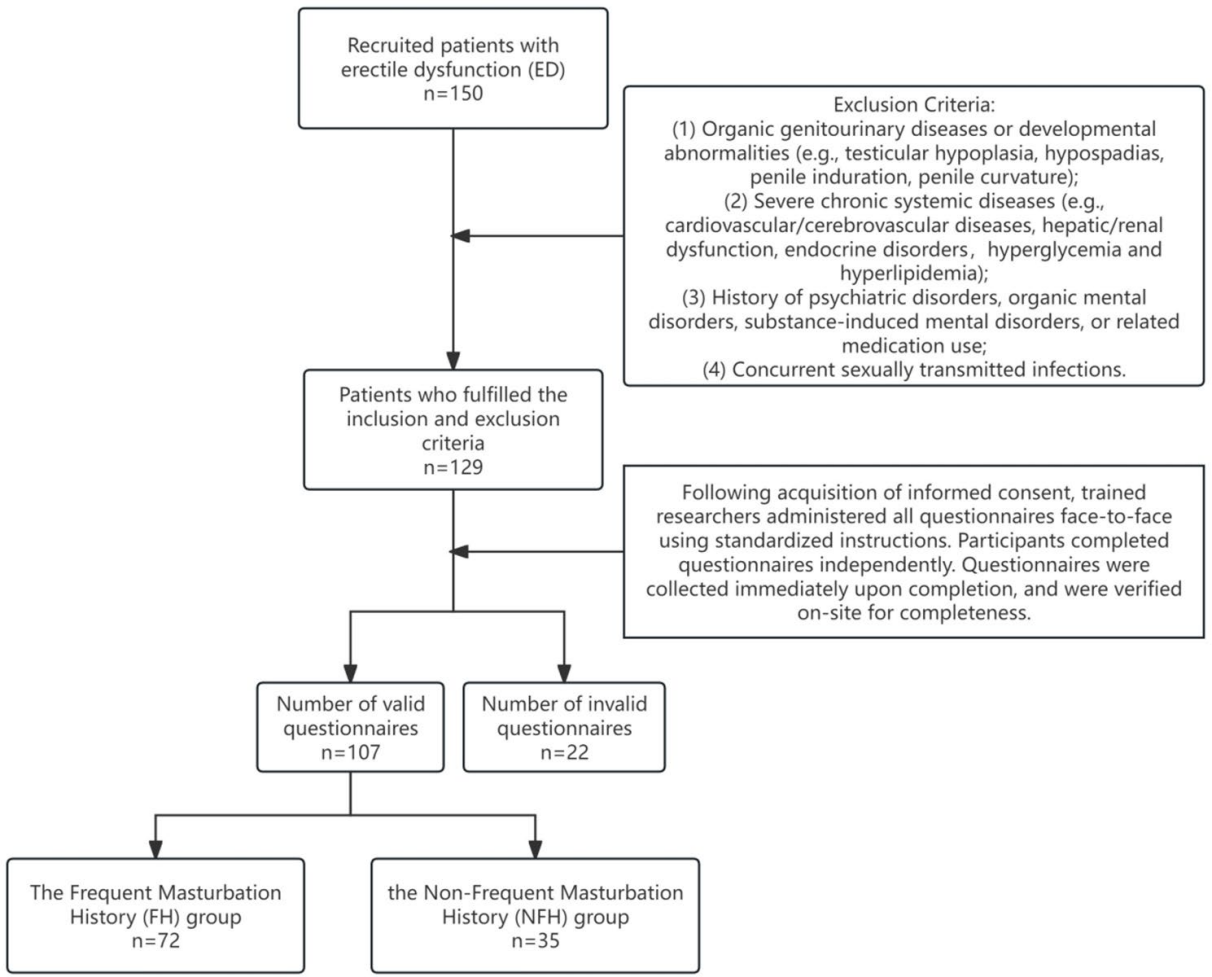
Flowchart of participant screening and enrollment

This cross-sectional observational study utilized consecutive sampling to recruit 107 patients diagnosed with psychogenic erectile dysfunction (pED) from the andrology outpatient clinic of a tertiary hospital in Xuzhou City between April 2024 and December 2024. The patient inclusion and exclusion procedures at each stage are detailed in Fig. 1. The study protocol was approved by the Ethics Committee of the Affiliated Hospital of Xuzhou Medical University (Approval No.: XYFY2024-KL411-01). Written informed consent was obtained from all participants.

### Inclusion and exclusion criteria

Inclusion Criteria:

1. Aged 18–65 years;
2. Diagnosed with pED based on medical history and relevant examinations; All patients with suspected psychogenic erectile dysfunction (pED) should undergo a comprehensive evaluation, including detailed history-taking, physical examination, and indicated instrumental investigations, to exclude potential organic causes. Erectile function was assessed by continuously measuring real-time penile tumescence and rigidity with a monitoring device(the RigiScan) during audiovisual sexual stimulation (AVSS). The evaluation was repeated after administering a phosphodiesterase-5 inhibitor. An adequate erectile response was defined as the maintenance of either: (1) mean rigidity ≥ 43.5% at the coronal sulcus concomitant with ≥ 50.5% at the penile base, or (2) mean rigidity > 60% at the penile base during full erection, with both criteria sustained for more than 8.75 min [1].
3. Having a stable sexual partner and at least one attempted sexual intercourse with regular sexual activity within the preceding 4 weeks;
4. Provision of written informed consent.

Exclusion Criteria:

1. Organic genitourinary diseases or developmental abnormalities (e.g., testicular hypoplasia, hypospadias, penile induration, penile curvature);
2. Severe chronic systemic diseases (e.g., cardiovascular/cerebrovascular diseases, hepatic/renal dysfunction, endocrine disorders, hyperglycemia and hyperlipidemia);
3. History of psychiatric disorders, organic mental disorders, substance-induced mental disorders, or related medication use;
4. Concurrent sexually transmitted infections.

### Study procedures

Data collection included gathering general information and assessing anxiety and depression levels.

#### Assessment instruments

General Information Questionnaire: Researcher-designed instrument capturing demographic and disease-related characteristics, including age, residence, smoking/alcohol history, disease duration, exercise habits, and masturbation history. A history of frequent masturbation was defined as self-reported masturbation frequency significantly exceeding normative levels, presence of compulsive behavior, or adverse impacts on psychological/physical well-being or daily functioning.

Erectile Function Assessment: Erectile function was quantified using the International Index of Erectile Function-5 (IIEF-5) questionnaire [11]. Scores were categorized as follows: ≤7 (severe ED), 8–11 (moderate ED), 12–16 (mild-to-moderate ED), 17–21 (mild ED), and 22–25 (no ED).

Anxiety and Depression Assessment: The Generalized Anxiety Disorder-7 (GAD-7) scale and the Patient Health Questionnaire-9 (PHQ-9) were employed for efficient clinical screening of anxiety and depressive symptoms, respectively [12, 13].

Psychological Resilience Assessment: The Connor-Davidson Resilience Scale (CD-RISC) was used to reliably quantify an individual’s capacity to adapt and recover from stress, trauma, or adversity, facilitating rapid identification of high-risk individuals with low resilience [14].

#### Data collection

Following acquisition of informed consent, trained researchers administered all questionnaires face-to-face using standardized instructions. Participants completed questionnaires independently. Questionnaires were collected immediately upon completion, and were verified on-site for completeness.

#### Statistical analysis

All conclusions of this study are grounded in empirical statistical analyses performed on retrospectively collected clinical data. Statistical analyses were performed using SPSS (version 26.0; IBM Corp., Armonk, NY, USA). Normality of continuous variables was assessed with the Shapiro-Wilk test. Normally distributed data are expressed as mean ± standard deviation (SD) and compared by independent samples *t*-tests; non-normally distributed data are summarized as median and interquartile range (IQR) and analyzed with the Mann-Whitney U test. Categorical variables are presented as number (percentage) and assessed using the χ² test or Fisher’s exact test, as appropriate. Statistical significance was defined as a two-sided *P* < 0.05.

## Results

### Comparison of baseline characteristics

Patients were categorized into two groups based on the presence or absence of a frequent masturbation history: the Frequent Masturbation History (FH) group and the Non-Frequent Masturbation History (NFH) group. Comparison of baseline characteristics demonstrated no statistically significant differences (*P* > 0.05) between the FH and NFH groups regarding disease duration, place of residence, smoking status, alcohol consumption, or regular exercise habits. However, statistically significant differences (*P* < 0.05) were observed between the two groups for age. See Table 1 for details.

**Table 1:** Comparison of baseline characteristics between FH and NFH groups

Variable	Total (*n* = 107)	FH (*n* = 72)	NFH (*n* = 35)	Statistic	*P*-value
IIEF-5,M (Q₁, Q₃)	14.00 (10.00, 17.00)	14.00 (10.00, 17.00)	16.00 (11.00, 19.00)	Z = -1.17	0.240
Disease duration (months), Mean ± SD	19.57 ± 31.21	22.42 ± 32.81	13.73 ± 27.14	*t* = 1.36	0.178
Age (years), M (Q₁, Q₃)	35.00 (29.00, 40.00)	32.00 (26.75, 36.25)	40.00 (36.00, 45.00)	Z = -4.83	< 0.001
Residence, n(%)				χ² = 0.57	0.452
Urban	68 (63.6)	44 (61.1)	24 (68.6)		
Rural	39 (36.4)	28 (38.9)	11 (31.4)		
Smoking, n(%)				χ² = 0.03	0.862
Yes	66 (61.7)	44 (61.1)	22 (62.9)		
No	41 (38.3)	28 (38.9)	13 (37.1)		
Alcohol use, n(%)				χ² = 0.02	0.877
Yes	47 (43.9)	32 (44.4)	15 (42.9)		
No	60 (56.1)	40 (55.6)	20 (57.1)		
Regular exercise, n(%)				χ² = 0.31	0.579
Yes	27 (25.2)	17 (23.6)	10 (28.6)		
No	80 (74.8)	55 (76.4)	25 (71.4)		

t: t-test, Z: Mann-Whitney test, χ²: Chi-square test

*SD* standard deviation, *M* Median, *Q₁* 1st Quartile, *Q₃* 3st Quartile

### Comparison of anxiety, depression, and psychological resilience scores

Comparison between participants with a history of frequent masturbation (FH group) and those without such a history (NFH group) demonstrated significant differences in psychological assessment scores. Anxiety scores were significantly higher in the FH group compared to the NFH group (Z = -2.17, *P* = 0.030). Depression scores were also significantly higher in the FH group than in the NFH group (Z = -3.01, *P* = 0.003). Conversely, psychological resilience scores were significantly lower in the FH group than in the NFH group (Z = -2.53, *P* = 0.011). See Table 2 for details.

**Table 2:** Comparison of anxiety, depression, and psychological resilience scores between FH and NFH groups

Variable	Total (*n* = 107)	FH (*n* = 72)	NFH (*n* = 35)	Statistic	*P*-value
GAD-7 Score, M (Q₁, Q₃)	8.00 (5.00, 11.50)	8.50 (5.75, 13.00)	6.00 (2.00, 10.00)	Z = -2.17	0.030
PHQ-9 Score, M (Q₁, Q₃)	6.00 (3.00, 10.00)	8.00 (4.75, 11.00)	5.00 (3.00, 7.50)	Z = -3.01	0.003
Psychological Resilience Score, M (Q₁, Q₃)	57.00 (47.00, 68.50)	55.00 (46.50, 63.00)	65.00 (52.50, 73.00)	Z = -2.53	0.011

Z: Mann-Whitney test

*M* Median, *Q₁* 1st Quartile, *Q₃* 3st Quartile

## Discussion

In recent years, research into the etiology of psychogenic erectile dysfunction (pED) has increasingly focused on the role of psychological and behavioral factors [15, 16]. Masturbation is a virtually universal component of human sexual behavior, the nature and consequences of which vary significantly with its frequency, context, and the individual’s psychological state. It is commonly regarded as part of healthy sexual exploration when devoid of intrapsychic conflict [17]. However, when masturbation becomes frequent, compulsive, and characterized by a loss of control (as observed in the FH subgroup), it is strongly correlated with psychological distress (e.g., anxiety and depression) and can serve as a maladaptive coping strategy [18]. Our findings align with this perspective, indicating that compulsive, high-frequency masturbation constitutes a clinically relevant node within the symptomatic network of psychogenic erectile dysfunction. Frequent masturbation may contribute to various organic conditions and can also exert a significant impact on psychological well-being. An International study suggests that excessive pornography consumption, masturbation, and/or promiscuity can lead to psychological distress and impairment in patients [19].

This study categorized pED patients into groups with (FH group) and without (NFH group) a history of frequent masturbation. We identified significant differences between the two groups in age, anxiety (GAD-7), depression (PHQ-9) scores, and psychological resilience scores. These findings provide important insights for understanding the heterogeneity of pED and developing individualized intervention strategies.

The mean age of patients in the FH group was significantly lower than in the NFH group, suggesting that frequent masturbation may be more prevalent among younger adults. This phenomenon could be related to physiological needs during the sexually active phase and psychosocial developmental stages: younger men face multiple pressures related to marriage, career, etc., and sexual behavior may become a means of anxiety relief [18]. A study from other countries indicates that feelings of shame, embarrassment, and guilt associated with masturbation are common among university students [20]. In contrast, Patients in advanced age groups may reduce reliance on high-frequency masturbation as an anxiety management tool, associated with a natural decline in sexual desire, greater knowledge about sexuality, or more mature stress-coping strategies [21, 22].

Anxiety and depression scores were significantly higher in the FH group compared to the NFH group, indicating a strong association between frequent masturbation and symptoms of anxiety and depression. Some international researchers have described the “Dhat syndrome,” where psychological problems arise from perceived semen loss. excessive masturbation, viewed as causing excessive semen loss, may contribute to anxiety and depression [23]. Due to its incentive systems, frequent masturbation might offer temporary stress relief [18]. However, over-reliance on it, potentially exacerbated by cultural stigma (such as beliefs related to “kidney deficiency” in traditional Chinese medicine), may intensify psychological conflict and trigger pED [17, 24]. Furthermore, traditional Chinese cultural disapproval of masturbation may foster cognitive distortions regarding “sexual inadequacy” in patients, amplifying the psychological burden associated with erectile failure [24]. In the course of administering the questionnaires, we frequently recorded spontaneous patient reports of post-masturbatory anxiety and guilt. However, the cross-sectional nature of this study limits the ability to establish causality between masturbation and anxiety/depression. Nevertheless, the strong associations observed offer empirical evidence supporting both competing theoretical perspectives. Future longitudinal studies are essential to determine temporal relationships and elucidate the underlying mechanisms.

Our data suggest that individuals in the FH group with lower psychological resilience may be more vulnerable to a vicious cycle: “masturbation to depression/anxiety to masturbation to erectile failure.” Conversely, the NFH group, potentially possessing higher resilience, may employ adaptive coping strategies (e.g., exercise, social support). This hypothesis aligns with Bandura’s self-efficacy theory, proposing that individuals with high resilience manage stress through diverse pathways, reducing dependence on a single sexual behavior [25]. Future research should investigate the potential mediating or moderating role of psychological resilience between the groups.

For FH group patients, clinical interventions should integrate behavioral modification with psychological support:

- Cognitive Behavioral Therapy (CBT): Help patients identify and modify irrational beliefs (e.g., “masturbation is harmful” or “erectile failure equates to loss of masculinity”) [26, 27].
- Mindfulness Training: Reduce excessive focus on sexual performance and alleviate anxiety-driven compulsive masturbation [28].
- Building Social Support Systems: Encourage partner involvement in therapy to reduce pressure during sexual interactions [29, 30].

Additionally, addressing the lack of culturally sensitive sex education in China is crucial. Public health initiatives should aim to destigmatize masturbation and promote scientifically grounded sexual health concepts [20].

## Study limitations

This cross-sectional study design precludes causal inference. Reliance on self-reported data introduces potential recall bias. Furthermore, the absence of biological markers (e.g., testosterone, cortisol levels) limits the depth of mechanistic exploration. This study also did not investigate specific masturbation techniques, precluding analysis of the relationship between technique and psychological state. A key limitation is that our study stratified participants based solely on masturbation frequency within a fixed recall period. This approach fails to capture dynamic changes in behavior, such as whether high-frequency patterns persisted, declined, or had recently escalated by follow-up. These distinct trajectories may differentially impact anxiety, depression, and erectile function, thereby potentially introducing unmeasured heterogeneity into the groups.

## Conclusion

Among patients with psychogenic erectile dysfunction (pED), a history of frequent masturbation is significantly associated with younger age, higher anxiety/depression scores, and lower psychological resilience scores. This finding highlights the necessity of incorporating assessments of sexual behavior patterns and psychological screening into the clinical evaluation of pED patients. For pED patients with a history of frequent masturbation, interventions focusing on enhancing psychological resilience and incorporating culturally adapted strategies may be more effective in breaking the “masturbation to depression/anxiety to masturbation to erectile dysfunction” vicious cycle [31]. This provides novel insights for improving the biopsychosocial health of Chinese males. Future research should employ longitudinal cohort designs incorporating biopsychosocial assessments to establish the temporal relationship between frequent masturbation and pED. Future longitudinal studies should track changes in masturbation frequencyover time.

## Data Availability

Data are available from the corresponding author upon reasonable request.

## Abbreviations

AVSS: Audiovisual sexual stimulation
CBT: Cognitive Behavioral Therapy
CD-RISC: The Connor-Davidson Resilience Scale
ED: Erectile dysfunction
FH: Frequent Masturbation History
GAD-7: Generalized Anxiety Disorder-7
IIEF-5: International Index of Erectile Function-5
IQR: Interquartile range
NFH: Non-Frequent Masturbation History
pED: Psychogenic erectile dysfunction
PHQ-9: Patient Health Questionnaire-9
SD: Standard deviation
